# Determination of Interleukin-6 and Tumor Necrosis Factor-alpha concentrations in Iranian-Khorasanian patients with preeclampsia

**DOI:** 10.1186/1471-2393-5-14

**Published:** 2005-11-01

**Authors:** J Tavakkol Afshari, N Ghomian, A Shameli, MT Shakeri, MA Fahmidehkar, E Mahajer, R Khoshnavaz, M Emadzadeh

**Affiliations:** 1 Immunogenetics department, Immunology Research Group, Bu-Ali Research Institute, Bu-Ali Sq., Mashhad University of Medical Sciences (MUMS), Mashhad, Iran; 2 Obstetrics & Gynecology department, Imam Reza Hospital, Imam Reza Sq., Mashhad University of Medical Sciences (MUMS), Mashhad, Iran; 3 Community medicine department, Medical school, Daneshgah St., Mashhad University of Medical Science (MUMS), Mashhad, Iran; 4 Department of Cardiology, Ghaem Hospital, Ahmad Abad St., Mashhad University of Medical Sciences (MUMS), Mashhad, Iran

## Abstract

**Background:**

Our objective was to determine the role of Interleukin-6 (IL-6) and Tumor Necrosis Factor-alpha (TNF-alpha), markers of immune activation and endothelial dysfunction, in patients with preeclampsia.

**Methods:**

Twenty four women with preeclampsia and eighteen antepartum normotensive pregnant women were recruited as controls. Serum levels of IL-6 and TNF-alpha were measured by enzyme-linked immunosorbent assay. We used independent-samples t test to assess the differences in the concentration of cytokines in preeclamptic patients and control subjects.

**Results:**

IL-6 levels [mean (S.D.)] were significantly higher in preeclamptic women [5.8 (4.85) pg/ml] compared to normal pregnant women [3.01 (2.45) pg/ml] (p = 0.02). There was no significant change in concentration of TNF-alpha in preeclamptic women [53.8 (30.0) pg/ml] compared to normal pregnant women [51.9 (33.8) pg/ml] (p > 0.1).

**Conclusion:**

The results of this study show that IL-6 as a pro-inflammatory cytokine is present in higher concentration in women with preeclampsia. The study was undertaken in women with established preeclampsia and it is not possible to determine whether the increased concentration of IL-6 is a cause or consequence of the disease. Furthermore, these findings suggest that serum TNF-alpha level is not associated with preeclampsia.

## Background

Preeclampsia is a critically important disease of pregnancy, one of the major causes of fetal and maternal morbidity and mortality throughout the world. In spite of its importance for public health, the etiology of preeclampsia has not yet been fully elucidated. Although the pathophysiology of preeclampsia is not understood completely, there is an interest in a possible link between inflammation and endothelial dysfunction [1] and between endothelial dysfunction and preeclampsia [2]. Cytokines have been described to play a major role in the pathogenesis of preeclampsia. Recent studies have demonstrated that cytokines – mediators of inflammatory response-may cause endothelial dysfunction through different mechanisms such as oxidative stress [3] and endothelial cell damage [4].

IL-6 is a proinflammatory cytokine produced by mononuclear phagocytes, endothelial cells, fibroblasts and T cells and has many functions and effects [5]. IL-6 is involved in immune activation, vascular wall function and modulation of TNF- production.

Recent studies have shown that amniotic fluid IL-6 is decreased in pregnancies complicated by preeclampsia and placental IL-6 production is decreased in these patients. These findings suggest a role for this cytokine in the pathophysiology of this disease. TNF-α is a potent modulator of immune and inflammatory responses that are produced by macrophages, lymphocytes and trophoblasts and contribute to the trophoblast growth and invasion. Its role in different pathologic conditions of pregnancy has been shown in recent studies. Increased amniotic fluid concentrations of TNF-α in patients with severe preeclampsia suggest a role for this cytokine in the pathophysiology of this disease. There are some studies in different populations that demonstrated the possible role of these cytokines in the pathophysiology of preeclampsia [6-10]. Therefore in the present study we measured their concentrations in serum of Khorasanian women (northeast of Iran) with preeclampsia, and compared them with concentrations found in normotensive control women.

Both IL-6 and TNF-α are expressed in adipose tissue [11,12] and *in vitro *release of TNF-α by adipocytes has been reported [13]. Among the known effects of these cytokines are inhibition of insulin signaling [14] and induction of both hypertriglyceridemia [15] and endothelial activation [16].

## Methods

This study was approved by the ethical committee of the Mashhad University of Medical Sciences (MUMS) and was conducted at Imam-Reza Hospital and Bu-Ali Research Institute. It was a cross-sectional study and preeclamptic patients were selected from those admitted to Imam-Reza Hospital between February 2004 and December 2004. Written informed consent was obtained from participating women. Twenty four patients with preeclampsia at 26–41 weeks' gestation were enrolled in this study. Preeclampsia was diagnosed according to the clinical criteria defined by the "International Society for the study of Hypertension in Pregnancy" [17]. All preeclamptic patients had blood pressure of at least 140/90 mmHg and proteinuria greater than (+) as assessed by a dipstick or 300 mg or greater in a 24-hour urine sample. None of the preeclamptic patients had underlying diseases such as chronic hypertension, diabetes mellitus, renal disease or urinary infection. None of these patients were in labor at the time of sampling. Some patients were prescribed hydralazine, dexamethazone or magnesium sulphate before sampling.

Eighteen antepartum normotensive healthy women at 26–39 weeks' gestation were included as control group. Maternal venous blood samples (5 ml) were collected from patient and control groups and were centrifuged at 3500 RPM for 10 minutes. Serum specimens were stored at -70°C in aliquots to avoid possible interference with assay results due to repeated freeze-thaw cycles.

Serum IL-6 and TNF-α concentrations were determined by commercially available high sensitivity indirect sandwich enzyme-linked immunosorbent assay (Bender MedSystems, Austria). Briefly, IL-6 or TNF-α present in the samples or standard binds to anti-IL-6 or anti-TNF-α monoclonal antibody adsorbed to the microwells. A biotin-conjugated monoclonal anti-IL-6 or anti-TNF-α antibody was added and binds to IL-6 or TNF-α captured by the first antibody. Following incubation unbound biotin-conjugated anti-IL-6 or anti-TNF-α is removed during a wash step. Streptavidin-HRP was added and binds to the biotin-conjugated anti-IL-6 or anti-TNF-α; following incubation unbound Streptavidin-HRP was removed during a wash step, and substrate solution reactive with HRP was added to the wells. A colored product was formed in proportion to the amount of IL-6 or TNF-α present in the sample. The reaction was terminated by addition of acid and absorbance was measured at 450 nm. A standard curve was prepared from seven IL-6 and TNF-α standard dilutions and IL-6 or TNF-α sample concentrations determined. The detection limit for IL-6 and TNF-α were 1.4 pg/ml and 3.83 pg/ml respectively. The overall inter-assay coefficient of variation for IL-6 and TNF-α were 5.2% and 6.9% respectively. All serum IL-6 and TNF-α analyses were performed at the same time, in the same batch, and in duplicate according to manufacturer's instructions. IL-6 and TNF-α serum levels are given as normal distribution. Independent-samples *t *test was used to compare the mean levels between patient and control groups. *P*-value < 0.05 were considered statistically significant.

## Results

**Figure 1:**
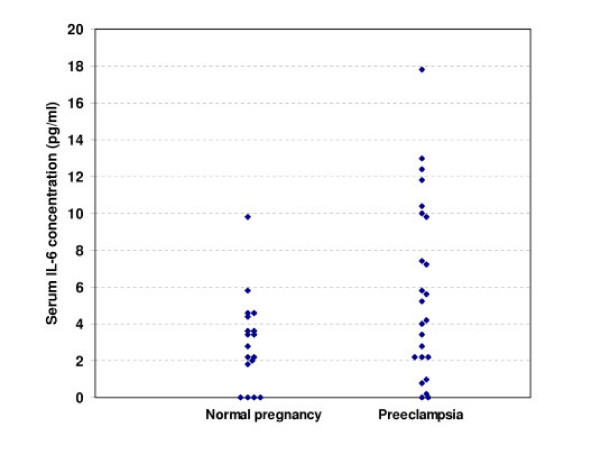
Serum IL-6 concentration (pg/ml) measured by ELISA in normal pregnant (n-18) and preeclamptic (n = 24) women.

A total of 24 women with preeclampsia and 18 women as controls were examined. The characteristics of women included in the study are presented in Table 1. There is no significance difference between two groups. Therefore the two groups were match for age, gestational age and gravity. IL-6 serum concentrations are depicted in Figure 1. IL-6 serum levels [mean (S.D.)] were significantly higher (p = 0.02) in preeclamptic women [5.8 (4.85)] compared with normal pregnant women [3.01 (2.45)].

**Figure 2:**
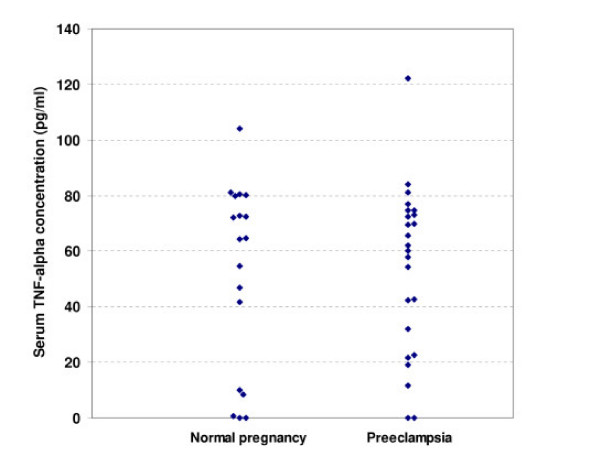
Serum TNF-alpha concentration (pg/ml) measured by ELISA in normal pregnant (n-18) and preeclamptic (n = 24) women.

TNF-α serum concentrations are depicted in figure 2. There was no significant Change in concentration of TNF-α serum levels in preeclamptic women [53.8 (30.0) pg/ml] compared to normal pregnant women [51.9 (33.8) pg/ml] (p > 0.1).

**Table 1:** 

	Normal pregnant (n = 18)	Preeclampsia (n = 24)	t and p
Age (years)	27.2 (5.8)	28.4 (4.9)	0.76, p > 0.1
Gestational age (weeks)	33.3 (3.7)	34.8 (4.3)	1.18, p > 0.1
Gravidity	2.3 (1.4)	2.2 (1.7)	0.65, p > 0.1

Data are presented as mean (S.D.).

## Discussion

Although the pathogenesis of preeclampsia is still unknown, immunologic and inflammatory causes may play an important role. IL-6 and other cytokines are important components of immune response, and therefore can participate in the immune aspects of the pathophysiology of this disease. Proinflammatory cytokines appear to be involved in cellular events that establish and maintain pregnancy [18]; however, their role has not yet been well defined.

Our data indicate that IL-6 concentrations were increased in the circulation of Iranian-Khorasanian preeclamptic patients compared with control women. This finding is consistent with reports by Greer et al in 1994 [6], Vince et al in 1995 [7], Kupferminc et al in 1996 [8], Munno et al in 1999 [9] and Teran et al in 2001 [10]. Endothelial dysfunction and increased endothelial permeability are the characteristics of the pathophysiology of preeclampsia. IL-6 may increase the permeability of endothelial cells [5] by changing the cell shape and rearrangement of intracellular actin fibers [19]. IL-6 can also reduce prostacyclin (PG I2) synthesis by inhibiting the cyclooxygenase enzyme [20]. It can increase the tromboxane A2 to prostacyclin ratio, an abnormality that happens in preeclampsia. IL-6 can also stimulate platelet-derived growth factor, a process seen in preeclampsia [5]. Oxygen free radicals can induce synthesis of IL-6 by endothelium [21]. Oxygen free radicals are implicated in the pathogenesis of preeclampsia, because they can cause endothelial damage, which leads to reduction in nitric oxide synthesis and prostaglandin balance disturbance.

TNF-α is another pro-inflammatory cytokine that its contribution in the pathogenesis of preeclampsia has been suggested in recent studies [8,10]. In healthy pregnant women, TNF-α is thought to modulate the growth and invasion of tropoblasts in maternal spiral arteries [22]. TNF-α may contributes to abnormal placental invasion [23], endothelial cell damage [4] and oxidative stress [3]. TNF-α can stimulates IL-6 production [24], since IL-6 inhibits TNF-α release [25]. In contrast to IL-6, no increased serum concentration of TNF-α was found in preeclamptic patients compared to control pregnant women. Several investigators [26-28] have reported that serum concentrations of TNF-α were significantly higher in the first and second trimester among pregnant women who subsequently developed preeclampsia compared to those in the control group. This finding is consistent with reports by Greer et al in1994 [6], Heyl et al in 1999 [28] and Ellis et al in 2001 [29]. Furthermore, the levels of IL-6 and TNF-α that we detected in the Khorsanian women studied were significantly higher than those reported for European and North American women [7,26,27]. Whether these differences are related to genetic (inflammatory response and L-arginine; NO pathway) and/or environmental factors (e.g., infection) remains to be determined. Several evidence links infection and inflammatory processes with preeclampsia [30,31]. The role of infection in the pathogenesis of preeclampsia is particularly relevant in developing countries, where the high incidence of chronic subclinical infection may contribute to the high incidence of preeclampsia.

Our findings support the hypothesis that immune activation is involved in preeclampsia and that IL-6 may participate in the abnormal immune response. This study was undertaken in women with established preeclampsia therefore, it cannot be determined whether the increase IL-6 was a cause or a consequence of the disease [7,10,26,27]. We cannot determine whether IL-6 is an active mediator in preeclampsia or a marker of immune activation, and it seems necessary to perform further studies, including longitudinal studies before the onset of preeclampsia, to elucidate the role of this cytokine in the pathogenesis of preeclampsia.

## Conclusion

The results of this study show that IL-6 as a pro-inflammatory cytokine is present in higher concentration in women with preeclampsia. The study was undertaken in women with established preeclampsia and it is not possible to determine whether the increased concentration of IL-6 is a cause or consequence of the disease. Furthermore, these findings suggest that serum TNF-α level is not associated with preeclampsia.

## Authors' contributions

JTA has participated in preparation and submission of the manuscript and given final approval of the version to be published. Also, conceived of the study, and participated in its design and coordination. NG participated in the diagnosis of patients with preeclampsia and in coordination of the study. AS participated in the diagnosis and selection of patients with preeclampsia. MTS participated in the design of the study and performed the statistical analysis. MAF carried out the immunoassays. MM participated in preparation of samples and performing ELISA for IL-6. RK: participated in drafting (English correction). ME participated in revising the manuscript critically for important intellectual content. All authors read and approved the final manuscript.

## Note

Table 1: Characteristics of women included in the study

## Pre-publication history

The pre-publication history for this paper can be accessed here:


